# Application of artificial neural networks to evaluate femur development in the human fetus

**DOI:** 10.1371/journal.pone.0299062

**Published:** 2024-03-13

**Authors:** Anna Badura, Mariusz Baumgart, Magdalena Grzonkowska, Mateusz Badura, Piotr Janiewicz, Michał Szpinda, Adam Buciński

**Affiliations:** 1 Department of Biopharmacy, the Ludwik Rydygier Collegium Medicum in Bydgoszcz, the Nicolaus Copernicus University in Toruń, Toruń, Poland; 2 Department of Normal Anatomy, the Ludwik Rydygier Collegium Medicum in Bydgoszcz, the Nicolaus Copernicus University in Toruń, Toruń, Poland; 3 Powiślański University in Kwidzyn, Kwidzyn, Poland; University of Life Sciences in Lublin, POLAND

## Abstract

The present article concentrates on an innovative analysis that was performed to assess the development of the femur in human fetuses using artificial intelligence. As a prerequisite, linear dimensions, cross-sectional surface areas and volumes of the femoral shaft primary ossification center in 47 human fetuses aged 17–30 weeks, originating from spontaneous miscarriages and preterm deliveries, were evaluated with the use of advanced imaging techniques such as computed tomography and digital image analysis. In order to ensure the data representativeness and to avoid introducing any hidden structures that may exist in the data, the entire dataset was randomized and separated into three subsets: training (50% of cases), testing (25% of cases), and validation (25% of cases). Based on the collected numerical data, an artificial neural network was devised, trained, and subject to testing in order to synchronously estimate five parameters of the femoral shaft primary ossification center, thus leveraging fundamental information such as gestational age and femur length. The findings reveal the formulated multi-layer perceptron model denoted as MLP 2-3-2-5 to exhibit robust predictive efficacy, as evidenced by the linear correlation coefficient between actual values and network outputs: R = 0.955 for the training dataset, R = 0.942 for validation, and R = 0.953 for the testing dataset. The authors have cogently demonstrated that the use of an artificial neural network to assess the growing femur in the human fetus may be a valuable tool in prenatal tests, enabling medical doctors to quickly and precisely assess the development of the fetal femur and detect potential anatomical abnormalities.

## 1. Introduction

In view of the immense bulk of data involved in contemporary medical research, the application of advanced analytical methods is imperative for modeling intricate nonlinear relationships. Artificial neural networks (ANNs) represent a tool capable of facilitating such modeling and serve as an alternative to conventional regression approaches [1, 2].

ANNs are biologically inspired by the human nervous system and have the capacity to emulate its structure and functioning. They comprise interconnected neurons that display characteristics analogous to those in the human brain [3]. ANNs are able to learn from the data provided, generalize knowledge to new instances, and perform parallel processing. Furthermore, ANNs demonstrate resilience to disturbances and errors [4, 5].

The utilization of artificial neural networks in medical research is especially relevant in the context of the current data explosion. Given their capacity to model complex nonlinear relationships, ANNs can unveil concealed patterns and relationships within medical data, thereby contributing to an enhanced comprehension of diseases, diagnoses, therapies, and medical outcome forecasting [6, 7].

ANNs are increasingly used as effective diagnostic tools for a variety of diseases and also for predicting therapeutic outcomes. Examples include ANN-based models used for the diagnosis of musculoskeletal disorders. In a study by Almhdie-Imjabbar, Nguyen [8], an ANN was involved to predict the progression of knee osteoarthritis based on radiological data. Furthermore, a study by Lu, Pareek [9] aimed to develop and validate a machine learning model that effectively predicted recurrent instability, progression to surgery, and the development of symptomatic osteoarthritis in patients with anterior shoulder instability (ASI). In a study by Wang, Song [10] using an ANN, clinical examination indicators were employed to predict interstitial lung disease (ILD) in patients with rheumatoid arthritis (RA).

This study attempts to assess the development of the femur in the human fetus using an artificial intelligence tool. The skeletal system is one of the earliest and rapidest developing systems during embryogenesis. As early as week 7 of the embryonic period, primary ossification centers appear in the shafts of long bones. Primary ossification centers play a substantial role in both gestational age assessment and the detection of potential developmental abnormalities. The process of ossification of the femur commences in its midshaft, progressing towards both its proximal and distal ends. Congenital malformations of lower limbs are frequently accompanied by intrauterine growth restriction.

Within the scope of this study, the authors designed, trained, and tested an ANN aimed at predicting the following five parameters of the femoral shaft primary ossification center in the human fetus: length, proximal, middle and distal transverse diameters, projection surface area and volume. For this purpose, two fundamental pieces of information were used: the gestational age and femur length.

## 2. Materials and methods

### 2.1. Computed tomography and digital image analysis

**Fig 1:**
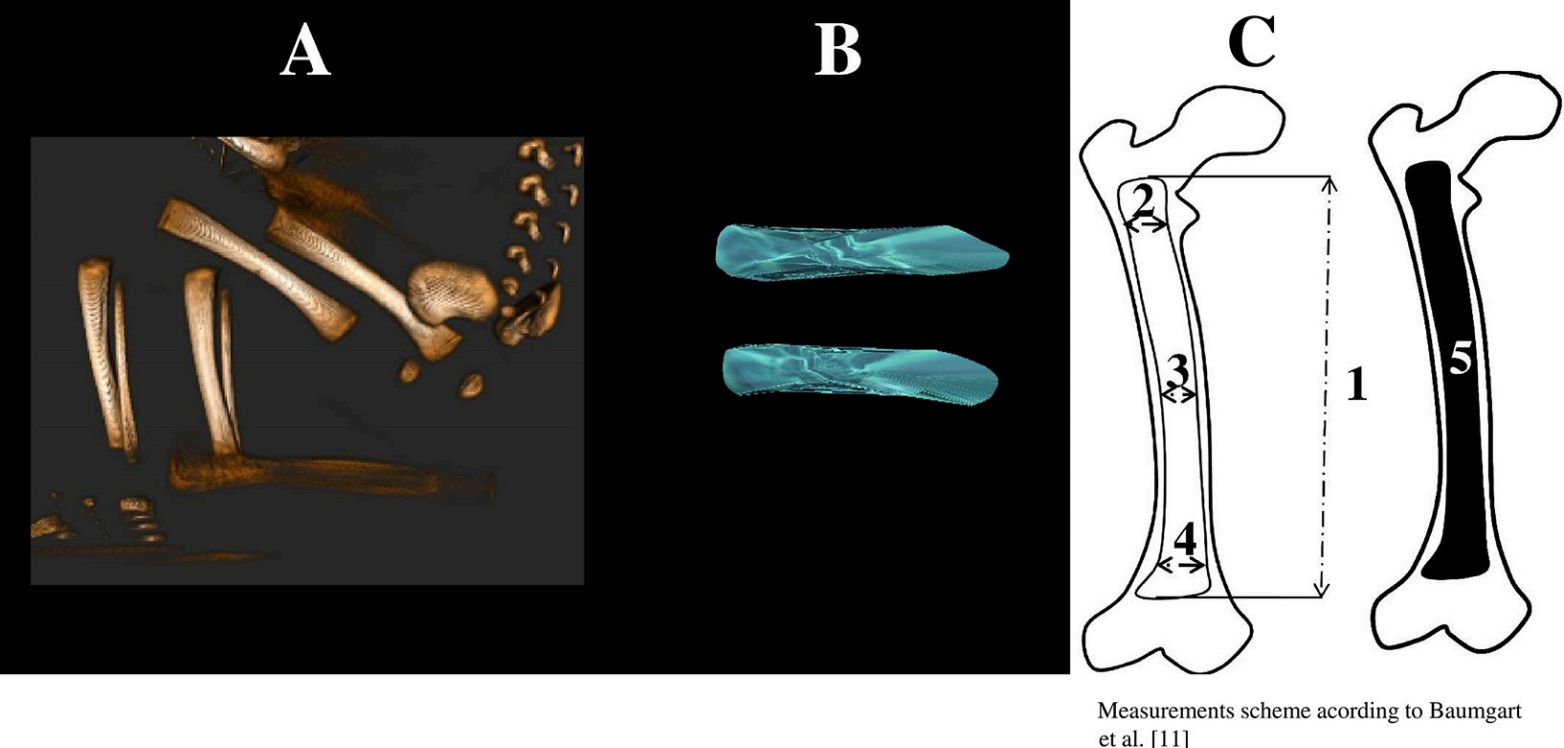
A–CT reconstruction of bones of a male fetus aged 22 weeks in the sagittal projection recorded in DICOM formats, B– 3D reconstruction of the right and left femoral shaft primary ossification centers assessed by Osirix 3.9. C–diagram showing measurements of the femoral shaft primary ossification center in the frontal plane: 1 –length; 2 –proximal transverse diameter; 3 –middle transverse diameter; 4 –distal transverse diameter; 5 –projection surface area.

The study encompassed 47 human fetuses (25 males and 22 females) aged 17–30 weeks of gestation. The fetuses were originated from spontaneous miscarriages and preterm deliveries, thus constituting the collection of the Department of Normal Anatomy. The fetuses were assessed for variability and explicit malformations by three independent observers. The gestational age was determined on the crown-rump length (CRL). Fetal scans in DICOM formats were performed using a Siemens-Biograph 128 mCT scanner, with intervals between successive sections of 0.4 mm. Linear, planar, and volumetric measurements for each femur were conducted using Osirix 3.9 software, according to the established protocol depicted in Fig 1. For each examined fetus, measurements of three transverse diameters, projection surface area and volume of the femoral shaft primary ossification center were carried out [11]. In spite of a cartilaginous stage of the femur, its contour was already clearly visible, thus enabling us to perform the quantitative evaluation of the femoral shaft primary ossification center [12, 13].

On both right and left sides the following six measurements of the femoral shaft primary ossification center were defined and performed:

1. length was defined as the distance between its proximal and distal boundaries in the frontal plane,
2. proximal transverse diameter was defined as the distance between its medial and lateral boundaries of its proximal region in the frontal plane,
3. middle transverse diameter was defined as the distance between its medial and lateral boundaries of its middle region in the frontal plane,
4. distal transverse diameter was defined as the distance between its medial and lateral boundaries of its distal region in the frontal plane,
5. projection surface area was calculated inside its contour in the frontal plane, and
6. volume was quantified by three-dimensional reconstructions, involving both spatial orientation and the attenuation of radiation by bony tissue.

### 2.2. Ethics statement

A retrospective study of medical records or archived samples has been reported. The Bioethics Committee of the Ludwik Rydygier Collegium Medicum in Bydgoszcz granted consent on May 10, 2011 (KB275). The morphometric examinations were carried out between January 1, 2020 and September 30, 2020 at the Department of Normal Anatomy of Ludwik Rydygier Collegium Medicum of Nicolaus Copernicus University in Toruń. Analysis of morphometric data using ANN was carried out in the period from December 20, 2022 to September 10, 2023. The fetuses have constituted the collection of the Department of Normal Anatomy. Furthermore, the authors did not have access to information that could identify individual participants either during or after the data collection process. All experiments were conducted in strict compliance with the pertinent named guidelines and regulations.

### 2.3. ANN analysis

The collected numerical data of the femoral shaft primary ossification center was analyzed using Statistica software (StatSoft, Inc., Tulsa, USA). A regression model with a multi-layer perceptron (MLP) grounded in a neural network was constructed, trained and tested. Since the statistical analysis for all the examined morphometric parameters revealed neither sex nor laterality statistically significant differences (P>0.05), all individual numerical data was aggregated for the entire study group, regardless of the sex and laterality.

The entire dataset, comprising numerical data of the femoral shaft primary ossification centers on both sides in the number of 94 (50 derived from 25 male fetuses and 44 derived from 22 female fetuses) was randomly separated into three subsets: training one (50% of cases), testing one (25% of cases), and validation one (25% of cases). These proportions were selected due to the necessity to provide an adequate amount of training data as well as independent sets for validation and testing. Randomization of the data was performed before separating into sets, thus ensuring a random and representative distribution. The model was based on two fundamental pieces of information − fetal femur length and gestational age–and predicted five specific parameters, as follows:

1. proximal transverse diameter of the femoral shaft primary ossification center,
2. middle transverse diameter of the femoral shaft primary ossification center,
3. distal transverse diameter of the femoral shaft primary ossification center,
4. projection surface area of the femoral shaft primary ossification center, and
5. volume of the femoral shaft primary ossification center.

**Fig 2:**
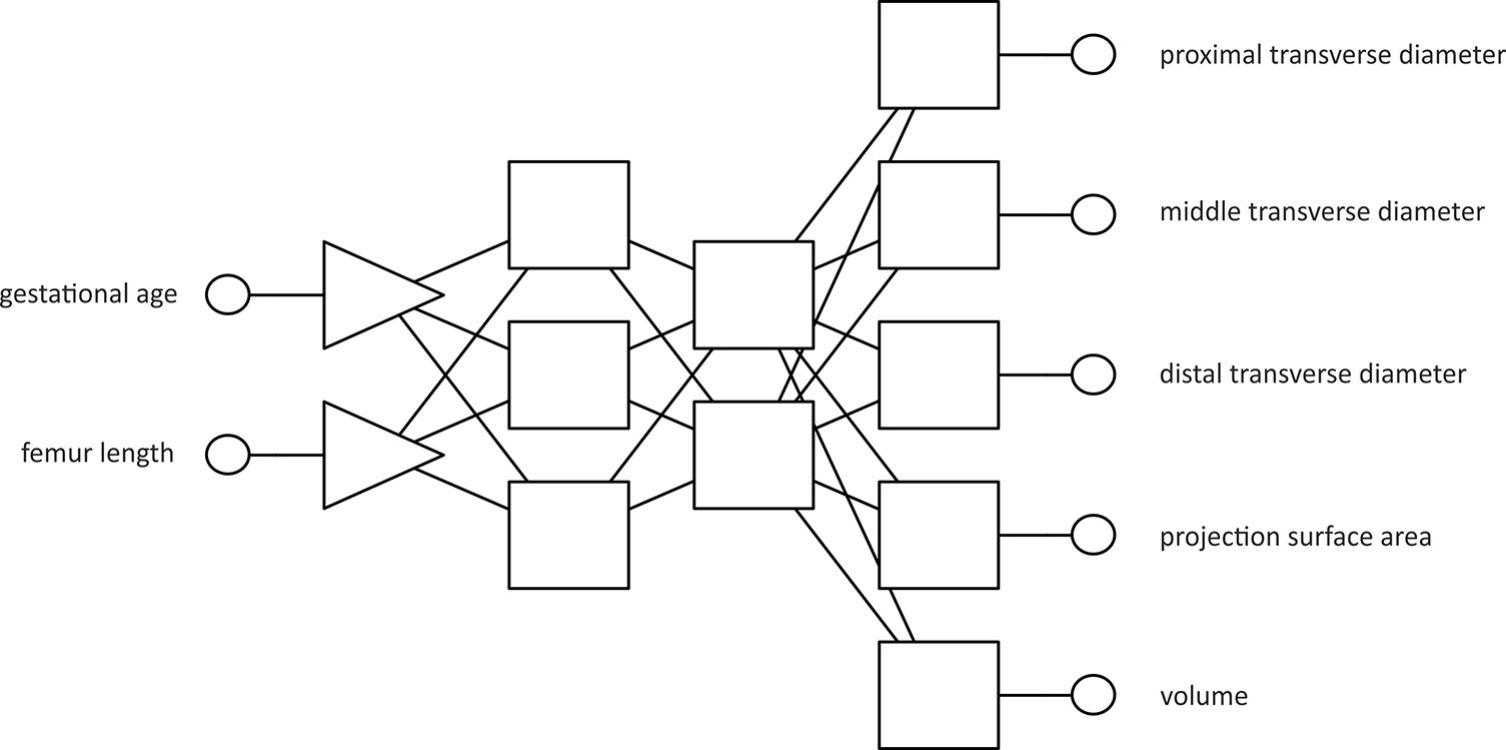
A layered structure of the MLP 2-3-2-5 regression model, with two input data and five predictions at the output.

The described regression model was based on a layered structure, adhering to the typical architecture of multi-layer perceptron (MLP) based on neural networks [5, 14, 15]. It consisted of four layers: an input layer, two hidden layers, and an output layer. In a multi-layer perceptron network, information flows from neurons in the input layer, through neurons in the hidden layers, to neurons in the output layer. In the input layer, each neuron corresponds to a single input variable that has been introduced into the model. Subsequently, two hidden layers were identified: the first one comprising three neurons and the second one with two neurons. The output layer included five neurons that generated the predicted responses of the model, representing the predicted values for the examined parameters of the femoral shaft primary ossification center (Fig 2). The transmission of signals between neurons of different layers is governed by activation functions, which are defined by mathematical relationships and determine the manner, in which information is processed [16, 17]. The applied activation functions in neurons of each network layer have been described in Table 1.

**Table 1:** Characteristics of the learning algorithms and activation functions employed in the MLP 2-3-2-5 model.

Learning algorithm	Activation function		
BP500, CG51b	Input layers	Hidden layers	Output layer
	linear function	hyperbolic functions	logistic function

The achievement of a high-quality model hinges on selecting the apposite network parameters. Adjusting these parameters results in reaching optimum performance and frequently involves an experimental process, in which various combinations of parameters are tested to find the best configuration for a given task. Thus, numerous tests were conducted on regression MLP models with diverse structures, varying numbers of neurons in hidden layers, and employing different activation functions.

The number of layers and neurons was experimentally determined by exploring different combinations. It should be emphasized that on one hand we circumvented excessive model complexity, which might result in overfitting, and on the other hand, we steered clear of too few neurons that might impede the learning process. Therefore, model testing began with a smaller number of neurons, which gradually increased while monitoring performance and preventing overfitting. Finally, after several attempts, an ANN model with a 2-3-2-5 architecture turned out to exhibit the best predictive performance.

The optimum configuration depends on the specific task, data type, and dataset size, thus making crucial both the experimentation and adjustment processes.

After selecting the optimum network architecture and activation functions, the model parameters were established in the form of weights connecting inputs to hidden neurons and those from hidden neurons to output neurons. The process of adjusting weights to ensure that the network effectively models the existing relationships between input data and output data is referred to as network training [18]. In the first stage, data from the training set are presented, which are used to train the neural network. For each input example, the network computes its output based on the given data, and then this output is compared to the expected output for that example. The difference between the actual and expected outputs is calculated as the prediction error. Subsequently, the network’s learning algorithms adjust the weights to minimize this error. This process is iterative, as the network repeats this procedure for many training examples, gradually refining the weights to enhance prediction accuracy. Presenting all cases from the training set once and modifying network parameters based on this process is referred to as a training epoch. In order to reduce model errors associated with either the arrangement or order of data, variable case presentation sequences were employed during successive epochs in training the neural network in this study. The effects of the training algorithm’s operation were monitored using a validation set. The final evaluation of the network’s quality was conducted with the use of an independent test set that was not employed during the stages of model training and validation [17, 18].

There are numerous methods for training neural networks, most of which involve more suitable weights, reached by iteration [15]. In the current study, network training was conducted in two stages. In the first stage, the backpropagation (BP) algorithm was applied for 500 epochs. Subsequently, in the second stage, additional 200 training cycles were conducted using the conjugate gradient (CG) method. Afterwards, the network was restored in the 51st training cycle when it reached the minimum validation set error, and no further decrease in error was observed. The learning rate and momentum for the BP algorithm were set to 0.01 and 0.3, respectively.

## 3. Results

### 3.1. Numerical data of the femoral shaft primary ossification center achieved by computed tomography and digital image analysis

Numerical data of the femoral shaft primary ossification center achieved by computed tomography and digital image analysis have been included in our previous original article [11]. As it turned out, the proximal, middle and distal transverse diameters of the femoral shaft primary ossification center increased proportionately: *y* = −3.579 + 0.368 × Age ± 0.529 (*R*^2^ = 0.88) for proximal transverse diameter, *y* = −1.105 + 0.187 × Age ± 0.309 *(R*^2^ = 0.84) for middle transverse diameter, and *y* = −2.321 + 0.323 × Age ± 0.558 (*R*^2^ = 0.83) for distal transverse diameter. Contrariwise, the remaining three parameters of the femoral shaft primary ossification center followed polynomial functions. Both its length and projection surface area followed the second-degree polynomial functions: *y* = 5.717 + 0.040 × (Age)^2^ ± 2.905 (*R*^2^ = 0.86) and *y* = −50.306 + 0.308 × (Age)^2^ ± 18.289 (R^2^ = 0.90), respectively. Furthermore, the volumetric growth of the femoral shaft primary ossification center modelled the third-degree polynomial function *y* = −91.458 + 0.390 × (Age)^3^ ± 92.146 (R^2^ = 0.88).

### 3.2. Model evaluation based on an ANN analysis

The subsequent stage of the study involved model evaluation. Various quality measures and errors were utilized for three datasets: the training set, the test set, and the validation set. Quality measures employed for the neural network under investigation included the ratio of standard deviations of prediction errors and the standard deviation of the output variable. This is referred to as the ratio of standard deviations. Furthermore, Pearson’s linear correlation coefficient (R) and the coefficient of determination (R^2^) were used to assess the model’s fit. The smaller values of the ratio of standard deviations indicated the better predictive model quality. The error was computed on the base of individual error values using the mean squared error (MSE) and function root mean squared error (RMSE).

**Fig 3:**
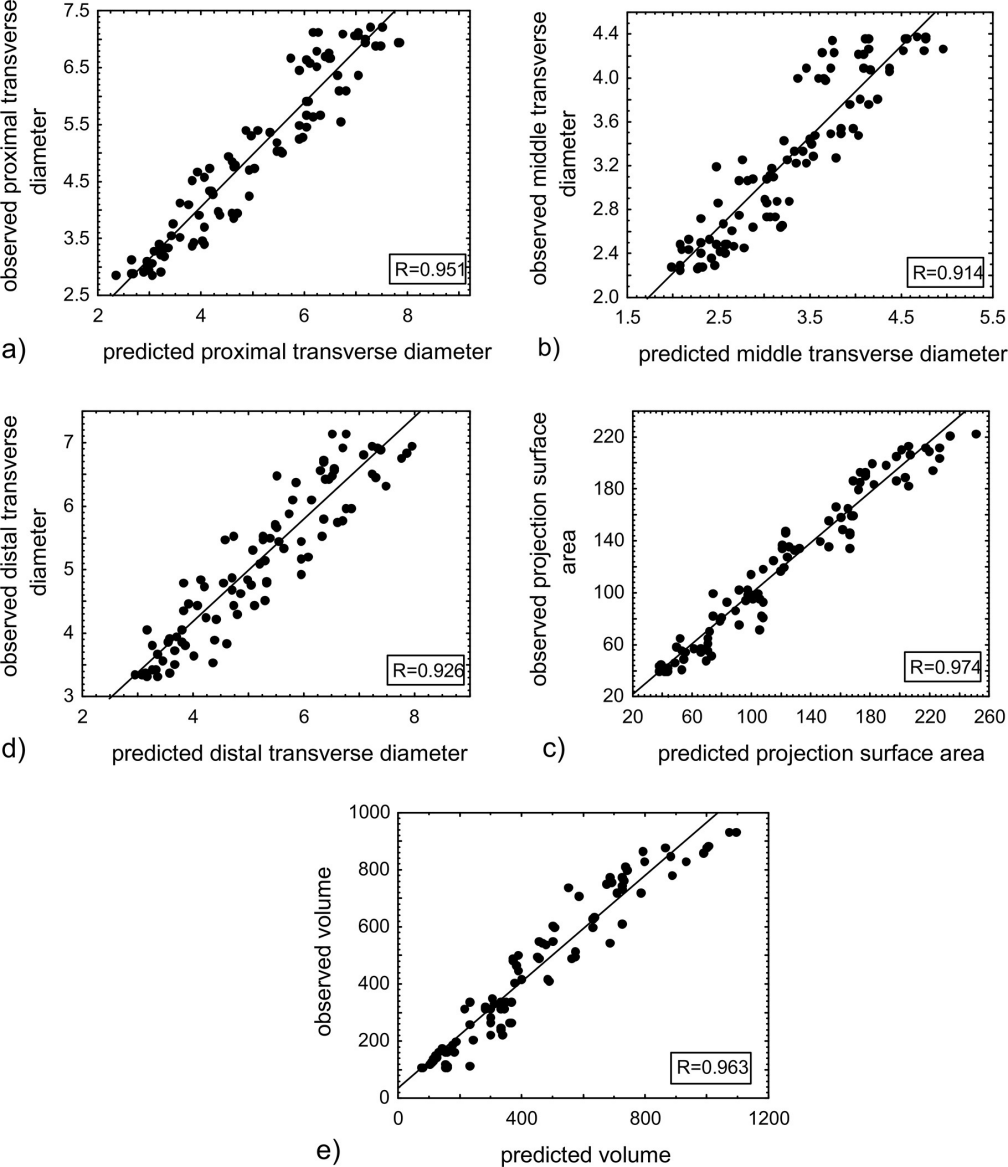
Results of the regression analysis. a) proximal transverse diameter of femoral shaft primary ossification center (R = 0.951); b) middle transverse diameter of femoral shaft primary ossification center (R = 0.941); c) distal transverse diameter of femoral shaft primary ossification center (R = 0.926); d) projection surface area of femoral shaft primary ossification center R = (0.974); e) volume of femoral shaft primary ossification center (R = 0.963).

**Fig 4:**
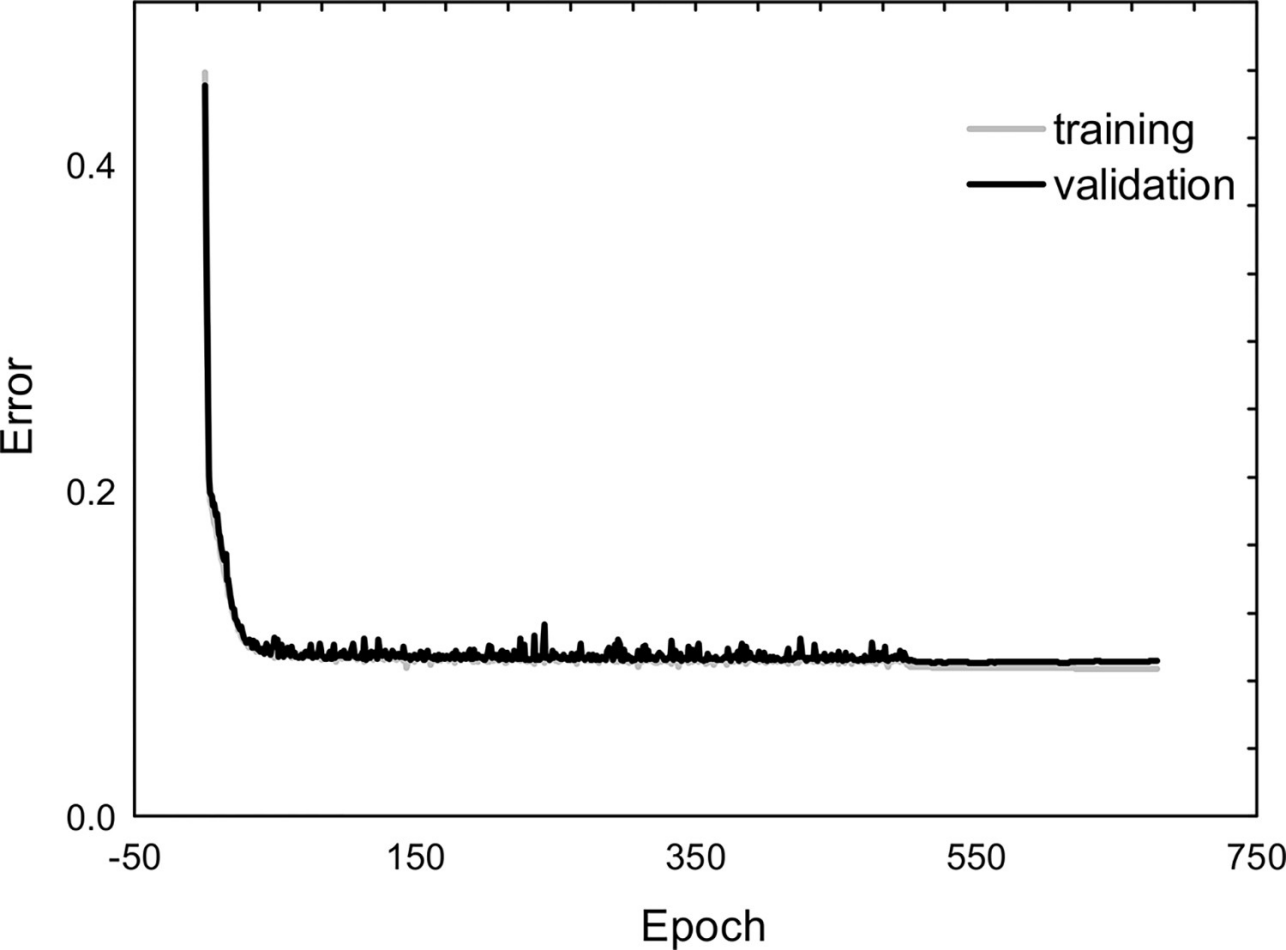
Learning charts for the training and validation sets of the MLP 2-3-2-5 model.

The findings of regression models were graphically presented as plots illustrating the correlation between predicted and observed values (Fig 3). A relatively good fit of the network to the analyzed problem was observed, thus confirming a high level of correlation coefficient (R) and the coefficient of determination (R^2^) for all predicted parameters (Table 2). Table 3 has presented results regarding the quality and error of the described predictive model. Meanwhile, Fig 4 has displayed the learning progress of the model across successive training epochs, thus enabling the monitoring of the neural network’s learning process and assessing whether the model is overfitting or excessively fitted to the data.

**Table 2:** Correlation coefficient results between observed and predicted values for all the parameters studied.

Parameters	Correlation coefficient (R)	Coefficient of determination (R^2^)
Proximal transverse diameter of femoral shaft primary ossification center	0.951	0.904
Middle transverse diameter of femoral shaft primary ossification center	0.914	0.835
Distal transverse diameter of femoral shaft primary ossification center	0.926	0.857
Projection surface area of femoral shaft primary ossification center	0.974	0.947
Volume of primary femoral shaft ossification center	0.963	0.925

**Table 3:** Quality measures of the MLP 2-3-2-5 model.

MLP 2-3-2-5	Ratio of standard deviations	Correlation coefficient (R)	Coefficient of determination (R^2^)	Mean square error (MSE)	Root mean square error (RMSE)
Training set	0.297	0.955	0.912	0.091	0.302
Test set	0.337	0.942	0.886	0.094	0.307
Validation set	0.302	0.953	0.909	0.091	0.302

## 4. Discussion

Many studies have focused on utilizing ANNs to predict various parameters in the human fetus. Coppedè [19] described an ANN as a useful tool supporting decision-making regarding both prenatal and perinatal care. Similarly, Feduniw, Golik [20] described the application of artificial intelligence, including an ANN, in screening studies for adverse perinatal conditions. Their research focused on the application of an ANN to assess the risk of pregnancy and childbirth complications, which could aid in the early detection and intervention in cases of high risk.

Another area of research has referred to estimating fetal weights due to ANN. A study conducted by Chen, Chen [21] emphasized the importance of an accurate assessment of the estimated fetal weight (EFW) in obstetrics. While regression methods are commonly employed, there is a need to enhance the accuracy of EFW due to the variability in birth weight and a distribution of deviation from the assumption of normality that may lead to the improper application of regression models. In response to this, researchers predicted EFW using a nonlinear ANN approach, which did not require the assumption of a normal distribution in the sample. The ANN models used in the study included a single hidden layer and were trained using the conjugate gradient algorithm that utilized ultrasound data such as biparietal diameter (BPD), occipitofrontal diameter (OFD), abdominal circumference (AC) and femur length (FL). The developed ANN model exhibited a high correlation coefficient between actual and estimated fetal body weights (R = 0.95; R^2^ = 0.89).

Clinical differentiation of congenital hip bone abnormalities is highly relevant during gynecological examinations as it influences the prognosis of lower limb development. The basic ultrasound assessment typically involves measurements of the ossified segment of femur shaft length, as cartilaginous parts of the epiphysial cartilages are not clearly visualized [22]. Any deviations in the size of the examined structures may contribute to the early detection of skeletal system anomalies that may involve either underdevelopment or agenesis of the femur. These anomalies may sporadically be accompanied by nervous system disorders or pathologies of the thorax and abdomen [23].

Hence, the application of an ANN for assessing the development of the femur in human fetuses appears to be a very promising approach. The concept of simultaneous estimation of five morphometric parameters of the femoral shaft primary ossification center based on simple information such as gestational age and femur length proved to be very effective. A two-stage training process was employed for the ANN model, utilizing two distinct learning algorithms: backpropagation and conjugate gradients. The artificial neural network for solving the regression problem, MLP 2-3-2-5, exhibited a high correlation between the actual values of all parameters of the femoral shaft primary ossification center and those predicted by the network, with the values of 0.955 for the training set, 0.942 for validation, and 0.953 for the test set. The error incurred by the discussed regression model was 0.091 for the training set, 0.094 for the validation set, and 0.091 for the test set. The graphical analysis of the described results indicates a robust response of the network to the posed problem. Furthermore, substantial correlations were obtained between observed and predicted values of individual parameters of the femoral shaft primary ossification center, such as its proximal transverse diameter (R = 0.951; R^2^ = 0.904), its middle transverse diameter (R = 0.914; R^2^ = 0.835), its distal transverse diameter (R = 0.926; R^2^ = 0.857), its projection surface area (R = 0.974; R^2^ = 0.947), and its volume (R = 0.963; R^2^ = 0.925).

The current findings indicate the promising potential of these methods in the field of both prenatal and perinatal care. Further research in this area may contribute to refining and advancing artificial intelligence-based tools that will aid in a considerably better understanding and assessing fetal health, as well as making informed clinical decisions.

Knowledge of the size of the examined femoral shaft primary ossification center holds significant potential utility in the diagnosis of skeletal dysplasias, which often manifest as disrupted growth or intrauterine growth restriction. To developmental anomalies of the femur belong proximal femoral focal deficiency, congenital short femur and Meyer dysplasia. In proximal femoral focal deficiency the affected femur corresponds to 35−50% of the length of a normal femur, while in congenital short femur, the affected femur has 40−60% of the length of a normal femur [24]. Meyer dysplasia results from delayed and uneven development of the primary ossification center at the proximal end of the femur.

In conclusion, this study demonstrates that the application of artificial neural networks may offer a promising approach for assessing femur development in prenatal tests. The ANN model developed by the authors show high accuracy in predicting femur parameters, based only on the two simple pieces of information such as gestational age and femur length. Further research and advancement in this field could contribute to both refining diagnostic tools and enhancing prenatal care. However, there are several issues related to our study limitations that should be considered in the context of further clinical research. Subsequent studies should address constraints associated with both the sample size and the representativeness of the data. Attention should be paid during the selection of the model architecture to the potential risk of model overfitting. The model should not be exaggeratedly complex for the available data, as this may lead to overfitting. The necessity of external validation is crucial, as a model developed on one clinical dataset may not be adequate enough for other clinical settings. Model verification in different clinical environments will be essential to confirm the model’s effectiveness. In conclusion, transitioning research to the clinical domain requires consideration of multiple factors, and the analysis and interpretation of results should be mindful of limitations, while simultaneously addressing the diversity and representativeness of clinical data. External validation in various clinical settings is a key component in confirming the model’s effectiveness.

## References

[1] Garcia-Vidal C, Sanjuan G, Puerta-Alcalde P, Moreno-García E, Soriano A. Artificial intelligence to support clinical decision-making processes. EBioMedicine. 2019;46:27–9. doi: 10.1016/j.ebiom.2019.07.019 31303500 PMC6710912

[2] Adlung L, Cohen Y, Mor U, Elinav E. Machine learning in clinical decision making. Med. 2021;2(6):642–65. doi: 10.1016/j.medj.2021.04.006 35590138

[3] Bishop CM. Neural networks for pattern recognition: Oxford university press; 1995.

[4] Haykin S. Neural networks: a comprehensive foundation: Prentice Hall PTR; 1998.

[5] Graupe D. Principles of artificial neural networks: World Scientific; 2013.

[6] Tong Z, Liu Y, Ma H, Zhang J, Lin B, Bao X, et al. Development, Validation and Comparison of Artificial Neural Network Models and Logistic Regression Models Predicting Survival of Unresectable Pancreatic Cancer. Front Bioeng Biotechnol. 2020;8:196. Epub 2020/04/02. doi: 10.3389/fbioe.2020.00196 ; PubMed Central PMCID: PMC7082923. 32232040 PMC7082923

[7] Mai RY, Zeng J, Meng WD, Lu HZ, Liang R, Lin Y, et al. Artificial neural network model to predict post-hepatectomy early recurrence of hepatocellular carcinoma without macroscopic vascular invasion. BMC Cancer. 2021;21(1):283. Epub 2021/03/18. doi: 10.1186/s12885-021-07969-4 ; PubMed Central PMCID: PMC7962237. 33726693 PMC7962237

[8] Almhdie-Imjabbar A, Nguyen KL, Toumi H, Jennane R, Lespessailles E. Prediction of knee osteoarthritis progression using radiological descriptors obtained from bone texture analysis and Siamese neural networks: data from OAI and MOST cohorts. Arthritis Res Ther. 2022;24(1):66. Epub 2022/03/10. doi: 10.1186/s13075-022-02743-8 ; PubMed Central PMCID: PMC8903620. 35260192 PMC8903620

[9] Lu Y, Pareek A, Wilbur RR, Leland DP, Krych AJ, Camp CL. Understanding Anterior Shoulder Instability Through Machine Learning: New Models That Predict Recurrence, Progression to Surgery, and Development of Arthritis. Orthop J Sports Med. 2021;9(11):23259671211053326. Epub 2021/12/11. doi: 10.1177/23259671211053326 ; PubMed Central PMCID: PMC8649098. 34888391 PMC8649098

[10] Wang Y, Song W, Wu J, Li Z, Mu F, Li Y, et al. Modeling using clinical examination indicators predicts interstitial lung disease among patients with rheumatoid arthritis. PeerJ. 2017;5:e3021. Epub 2017/03/01. doi: 10.7717/peerj.3021 ; PubMed Central PMCID: PMC5322753. 28243535 PMC5322753

[11] Baumgart M, Wiśniewski M, Grzonkowska M, Badura M, Małkowski B, Szpinda M. Quantitative anatomy of the primary ossification center of the femoral shaft in human fetuses. Surg Radiol Anat. 2017;39(11):1235–42. Epub 2017/04/27. doi: 10.1007/s00276-017-1861-8 ; PubMed Central PMCID: PMC5644710. 28444434 PMC5644710

[12] Chano T, Matsumoto K, Ishizawa M, Morimoto S, Hukuda S, Okabe H, et al. Analysis of the presence of osteocalcin, S-100 protein, and proliferating cell nuclear antigen in cells of various types of osteosarcomas. Eur J Histochem. 1996;40(3):189–98. Epub 1996/01/01. . 8922947

[13] Duarte WR, Shibata T, Takenaga K, Takahashi E, Kubota K, Ohya K, et al. S100A4: a novel negative regulator of mineralization and osteoblast differentiation. J Bone Miner Res. 2003;18(3):493–501. Epub 2003/03/07. doi: 10.1359/jbmr.2003.18.3.493 . 12619934

[14] Hippert HS, Pedreira CE, Souza RC. Neural networks for short-term load forecasting: A review and evaluation. IEEE Transactions on power systems. 2001;16(1):44–55.

[15] Weiss R, Karimijafarbigloo S, Roggenbuck D, Rödiger S. Applications of Neural Networks in Biomedical Data Analysis. Biomedicines. 2022;10. doi: 10.3390/biomedicines10071469 35884772 PMC9313085

[16] Lederer J. Activation Functions in Artificial Neural Networks: A Systematic Overview. ArXiv. 2021;abs/2101.09957.

[17] Dongare A, Kharde R, Kachare AD. Introduction to artificial neural network. International Journal of Engineering and Innovative Technology (IJEIT). 2012;2(1):189–94.

[18] Abraham A. Artificial neural networks. Handbook of measuring system design. 2005.

[19] Coppedè F. The genetics of folate metabolism and maternal risk of birth of a child with Down syndrome and associated congenital heart defects. Front Genet. 2015;6:223. Epub 2015/07/15. doi: 10.3389/fgene.2015.00223 ; PubMed Central PMCID: PMC4479818. 26161087 PMC4479818

[20] Feduniw S, Golik D, Kajdy A, Pruc M, Modzelewski J, Sys D, et al. Application of Artificial Intelligence in Screening for Adverse Perinatal Outcomes-A Systematic Review. Healthcare (Basel). 2022;10(11). Epub 2022/11/12. doi: 10.3390/healthcare10112164 ; PubMed Central PMCID: PMC9690973. 36360505 PMC9690973

[21] Chen C-Y, Chen Y-F, Chen H-Y, Hung C-T, Shi H-Y. Artificial Neural Network and Cox Regression Models for Predicting Mortality after Hip Fracture Surgery: A Population-Based Comparison. Medicina. 2020;56(5):243. doi: 10.3390/medicina56050243 32438724 PMC7279348

[22] Nemec U, Nemec SF, Weber M, Brugger PC, Kasprian G, Bettelheim D, et al. Human long bone development in vivo: analysis of the distal femoral epimetaphysis on MR images of fetuses. Radiology. 2013;267(2):570–80. Epub 2013/02/09. doi: 10.1148/radiol.13112441 . 23392423

[23] Ring PA. Congenital abnormalities of the femur. Arch Dis Child. 1961;36(188):410–7. Epub 1961/08/01. doi: 10.1136/adc.36.188.410 ; PubMed Central PMCID: PMC2012716. 13741753 PMC2012716

[24] Gillespie R, Torode IP. Classification and management of congenital abnormalities of the femur. J Bone Joint Surg Br. 1983;65(5):557–68. Epub 1983/11/01. doi: 10.1302/0301-620X.65B5.6643558 . 6643558

